# The effectiveness of the serious game “Broodles” for siblings of children with intellectual disabilities and/or visual impairment: study protocol for a randomized controlled trial

**DOI:** 10.1186/s13063-023-07358-1

**Published:** 2023-05-17

**Authors:** Linda K. M. Veerman, Agnes M. Willemen, Suzanne D. M. Derks, Anjet A. J. Brouwer-van Dijken, Paula S. Sterkenburg

**Affiliations:** 1 Department of Clinical Child and Family Studies; LEARN!; Amsterdam Public Health, Vrije Universiteit Amsterdam, Amsterdam, Van der Boechorststraat 7, 1081 BT the Netherlands; 2 Brussenboek.nl, Bilthoven, the Netherlands; 3 Sibling Carers Community BRUS, Utrecht, the Netherlands; 4 Bartiméus, Doorn, Oude Arnhemse Bovenweg 3, 3941 XM the Netherlands

**Keywords:** Siblings, Quality of life, Psychosocial well-being, Serious game, Intellectual disability, Visual impairment

## Abstract

**Background:**

Siblings of children with disabilities also need support. However, there are only a few evidence-based interventions for these siblings. The current study aims to assess the effectiveness of a newly developed serious game for young siblings of children with intellectual disability (ID) and/or visual impairment (VI). This serious game is hypothesized to improve sibling’s quality of life, adjustment to their brother’s or sister’s disability, and multiple aspects of psychosocial well-being.

**Methods:**

The intervention consists of a serious game called “Broodles” (in Dutch: “Broedels”) that helps children to recognize and deal with thoughts, feelings, and difficult situations. The game consists of eight 20-minute levels that all have the same structure with eight game elements. Each level addresses a domain of sibling quality of life and combines animations, mini-documentaries, fun mini-games, and multiple-choice questions. In addition to the game, siblings make a worksheet after playing each level. In order to support the child, the parents or caregivers receive a short brochure with information and tips. The effectiveness of the intervention will be investigated among a sample of 154 children aged 6–9 years and their parents or caregivers, using a two-arm parallel RCT design. The experimental group will play the serious game “Broodles” over a period of 4 weeks, whereas the control group will be placed on a waiting list. Assessments will take place at three time points: pre-test (week 1), post-test (week 5), and follow-up (weeks 12–14). At each timepoint, children and parents will complete several questionnaires on quality of life and different aspects of psychosocial well-being. In addition, children will make drawings to assess the sibling relationship. Next to that, parents and children will answer closed and open-ended questions about the sibling adjustment to their brother or sister’s disability. Finally, parents and children will evaluate the serious game through closed and open-ended questions.

**Discussion:**

This study contributes to the knowledge about sibling interventions and serious games. Additionally, if the serious game is proven to be effective, it will be a readily available, easily accessible, and free of charge intervention for siblings.

**Trial registration:**

ClinicalTrials.gov, NCT05376007, registered prospectively on April 21, 2022.

## Administrative information

Note: the numbers in curly brackets in this protocol refer to SPIRIT checklist item numbers. The order of the items has been modified to group similar items (see http://www.equator-network.org/reporting-guidelines/spirit-2013-statement-defining-standard-protocol-items-for-clinical-trials/).

**Table:** 

Title {1}	The Effectiveness of the Serious Game “Broodles” for Siblings of Children with Intellectual Disabilities and/or Visual Impairment: Study protocol for a Randomized Controlled Trial Running head: The Effectiveness of the Serious Game “Broodles”
Trial registration {2a and 2b}.	ClinicalTrials.gov, NCT05376007, registered prospectively on April 21, 2022
Protocol version {3}	Version 1, May 19, 2022 Version 2, May 2, 2023
Funding {4}	The study is funded by the Academic Lab ‘Social Relations and Attachment’ (project number 641001101), which is funded by The Netherlands Organization for Health Research and Development ZonMw, Den Haag, the Netherlands.
Author details {5a}	Linda K. M. Veerman, email: l.k.m.veerman@vu.nl, ORCID: https://orcid.org/0000-0003-0306-1661 Agnes M. Willemen, email: a.m.willemen@vu.nl ORCID: https://orcid.org/0000-0002-0912-2608 Suzanne D. M. Derks, email: s.d.m.derks@vu.nl ORCID: https://orcid.org/0000-0001-7656-6455 Anjet A. J. Brouwer - van Dijken, email: anjetxt@gmail.com Paula S. Sterkenburg, email: p.s.sterkenburg@vu.nl ORCID: https://orcid.org/0000-0001-6014-7539 ^1^ Department of Clinical Child and Family Studies; LEARN!; Amsterdam Public Health, Vrije Universiteit Amsterdam, Van der Boechorststraat 7, 1081 BT Amsterdam. ^2^ Bartiméus, Oude Arnhemse Bovenweg 3, 3941 XM Doorn, the Netherlands. ^3^ Brussenboek.nl [1] ^4^ Sibling carers community BRUS [2] ^*^ Corresponding author: l.k.m.veerman@vu.nl
Name and contact information for the trial sponsor {5b}	Department of Clinical Child and Family Studies, Vrije Universiteit Amsterdam, Van der Boechorststraat 7, 1081 BT Amsterdam. p.s.sterkenburg@vu.nl
Role of sponsor {5c}	There are no obligations towards the sponsor other than reporting about the study and study results.

## Introduction

### Background and rationale {6a}

The sibling relationship is both special and important, as well as the longest in our lives [3]. When one of the siblings has a disability, it affects the lives of all siblings in the family and leads to both positive experiences and difficulties. Siblings can for example experience feelings of worry, guilt, embarrassment, or jealousy, but also proudness and love for their brother or sister with a disability [4]. On top of that, siblings take into account their brother’s or sister’s special needs, which can result in not being able to participate in social activities with friends or family [5]. For example, they cannot do certain activities because these make their brother or sister upset or because their brother or sister is not able to do these activities. Siblings could use support with these challenges [6]. However, care and support mostly focus on the child with a disability, and policies on support sources for siblings are insufficient [7].

Previous studies have reported that siblings of children with a disability or other chronic conditions show more psychosocial problems than siblings of typically developing children [8, 9]. For example, they show more emotional problems and problems in everyday functioning (e.g., school functioning and social relationships) and are more likely (almost three times) to experience “significant functional impairment” than siblings of healthy children [8]. However, other studies find that these differences can be contributed to co-occurring risk factors, for instance, socio-economic status and single parent household [10, 11], sibling relationship [12], and negative behavior of the brother or sister with a disability [6, 13, 14]. Despite, qualitative studies show that having a brother or sister with a disability does impact the quality of life and well-being of siblings [15].

For this reason, it has been recommended to offer early interventions to siblings to prevent problems [6, 8]. In an integrated review of 28 quantitative and qualitative studies on the experiences of siblings of children with Down syndrome or autism spectrum disorder (ASD), a few implications for supporting siblings were drawn based on the reported experiences [6]. These include the following: help siblings to deal with negative behavior of their brother or sister, let them experience that they are not the only one having certain emotions, give them insights into how they can engage in play with their brother or sister, and help them to explain the condition of their brother or sister to peers.

Several interventions and support programs for siblings of children with different kinds of disabilities or conditions have been developed and studied, showing small effects for example on well-being [16]. However, mixed results were found in the different studies, and most of these studies had small sample sizes and did not include a control group. Therefore, it was concluded that more RCTs are needed to be able to identify which aspects of interventions have positive impact on siblings’ wellbeing, which outcomes should be targeted with the interventions, and what is desirable when determining the target group [16]. Moreover, most studied interventions included support groups with live sessions for both siblings and parents [17, 18], making them costly and time-consuming for families that already have high care burdens.

A low-cost and easy to access intervention type is a serious game, a computer game that addresses educational and therapeutic goals in a fun and playful manner. Serious games have benefits over regular mental-health interventions, because they have high feasibility, and are appealing and engaging [19]. In a meta-analysis [20], it was found that serious games for children show small but significant effects in improving mental-health or health-related behavior. Another meta-analysis showed that serious games can improve social(-emotional) adaptative and cognitive skills in children with ID or ASD [21]. However, the authors of both studies stress that more research is needed in order to be able to conclude that serious games are beneficial in improving mental health or specific skills. An example of an effective serious game is “See,” developed for children aged 6–8 years with a visual impairment [22]. Next to positive experiences of the children, playing the game had a significant positive effect on academic self-concept and social inclusion of the children compared to a control group.

The aim of the current trial is to study such a serious game which we have developed for young siblings of children with disabilities to support their quality of life and psychosocial well-being. When developing and studying a serious game, three main aspects are of importance, which have been taken into account in this study as well. Firstly, in contrast to sibling support groups, in serious games it is highly important to identify a specific target group and focus on their specific needs by involving them in the development process through co-creation [23, 24]. In the current study, selecting this target group was done based on age and type of condition of the brother or sister. Considering age, middle childhood (6–12 years) is an often targeted age group for sibling interventions [18], which is appropriate, because from this age, children start to develop important skills in reasoning, that are necessary to evaluate and learn to cope with situations, gain self-concept, and become able to verbalize thoughts and emotions [25]. Furthermore, few interventions have been developed for children aged 6 to 8 years old, or specifically for siblings of children with ID, and none have focused on children with visual impairment (VI) [16].

Secondly, the content of the serious game needs to have a strong empirical basis [20]. In the current serious game, the content was based on several studies that argue that it can be helpful for siblings to focus on how to handle experienced emotions and difficult situations [6, 18], and to offer knowledge that helps the sibling to better understand their brother or sister [16]. In particular, in the study of Moyson and Roeyers [15], the experiences siblings of children with intellectual disabilities (ID) describe are conceptualized in nine domains which are relevant to sibling quality of life. These domains, which are embedded in the current serious game, include the following: “joint activities,” “mutual understanding,” “private time,” “acceptance,” “forbearance,” “trust in well-being,” “exchanging experiences,” “social support,” and “dealing with the outside world.”

Thirdly, in studying the effect of a serious game, specific outcomes should be targeted and measured [20]. In the current study, the overall aim of the intervention was to improve sibling quality of life and adjustment through targeting different aspects of psychosocial well-being. These secondary outcomes were selected based on the results from previous intervention studies [16–18, 26] and outcomes that were found to influence sibling well-being or quality of life [10, 12, 27, 28]. The serious game includes elements that focus on improving siblings’ adjustment to the disability, coping skills and self-worth, and increasing the feeling of being supported. In addition, the intervention stimulates parents and siblings to talk and share their feelings about the brother or sister with a disability, which may increase parent-child closeness. Improvement on these outcomes has been found in two different onsite sibling group interventions [26, 29], and it is important to assess if an online individual intervention can successfully target these outcomes as well. Next to that, the game aims to help siblings to better understand their brother or sister, which could lead to a warmer sibling relationship. A study among siblings of children with ASD showed that better perspective taking abilities were associated to more positive affect towards the brother or sister [30]. Because the intervention includes a manual for parents, and parental factors, such as parental stress [10], have been found to be of influence on sibling wellbeing, it is also of interest to explore the effect of the intervention on the parent. Parenting self-efficacy [31] will therefore be investigated in this study, because it has often been targeted in interventions for parents of children with disabilities [32], and it was found to be a predictor of family adjustment in families with a child with a disability [33].

In addition to the beforementioned three aspects, it is important to evaluate how well the serious game can be implemented in practice (social validity) and which family characteristics can moderate the effectiveness [34]. Therefore, the following family characteristics that were implied to be of importance in previous sibling research have been selected as moderators for the current study: negative behavior of the brother or sister with a disability [14], sibling relationship [12], and parent-child relationship [7].

### Objectives {7}

In the present study, the main aim is to investigate whether the serious game is more effective in improving sibling quality of life and adjustment to the disability of the brother or sister, compared to waitlist control group. Secondly, it will be investigated whether the serious game is more effective compared to the waitlist control group in improving different aspects of psychosocial well-being. These include the following: (1) siblings’ self-esteem, (2) siblings’ perceived social support, (3) sibling relationship with the brother or sister with a disability, (4), siblings’ coping skills, and (5) parent-child relationship. Thirdly, the social validity of the serious game will be investigated in order to evaluate the desirability, applicability, and subjective evaluation of the game. Finally, exploratory analyses will be executed to investigate possible moderators and the effect of the intervention on parenting self-efficacy.

### Trial design {8}

The effectiveness of the serious game will be examined using a parallel superiority randomized controlled trial (RCT) design with quantitative and qualitative assessments on three measuring moments: pre-test (T0), post-test (T1; 5 weeks after pre-test), and follow-up (T2; 6–8 weeks after post-test). The children will be randomly assigned to two groups of equal size using stratified block randomization: an experimental group that will play the serious game and a waitlist control group.

The methods of this study are reported according to SPIRIT 2013 Explanation and Elaboration: Guidance for protocols of clinical trials [35].

## Methods: participants, interventions and outcomes

### Study setting {9}

The study is conducted in one center, which is the Department for Clinical Child and Family Studies at the Vrije Universiteit Amsterdam. Siblings and parents or caregivers (hereafter indicated as “parents”) of children with ID and/or VI living in the Netherlands and Belgium (only the Dutch-speaking part, Flanders) will be recruited from the general population. The study procedures will take place at the homes of the participants.

### Eligibility criteria {10}

Dutch-speaking siblings between the age of 6 and 9 years and 11 months who live in the Netherlands or Flanders with a brother or sister with ID and/or VI will be included. The included siblings live in the same house as their brother or sister, at least part of the time. The brother or sister with a disability can have different levels of ID, different levels of VI, and different comorbid conditions and either attend regular education, special education, or day-care facilities. Excluded from participation are siblings that have a disability or severe illness; siblings with parents who have a disability or severe illness; and siblings of children who live in home-care facilities on a full-time basis. Only one sibling and one parent per household will be included in the study. The family members can decide who will participate.

### Who will take informed consent? {26a}

When inclusion criteria are met, the parent will receive the participant information letter by email. A simplified child-version of the information letter will also be sent to the parent, so that the parent can discuss the research procedure with the sibling. When the parent and sibling want to participate in the study, the parent will be asked to sign and return two consent forms: one in behalf of the sibling and one for their own participation in the research. The consent form for the sibling’s participation also needs to be signed by the other parent that has custody or by the legal guardian. The signed informed consent forms can be given to the research assistant at the beginning of the pre-test assessment. The parent and child can ask questions about the study procedure to the research assistant before handing over the informed consent form. This research assistant will also sign the informed consent form in order to declare that they have fully informed the participant.

### Additional consent provisions for collection and use of participant data and biological specimens {26b}

Specific consent is obtained for making audio recordings from the answers of the children to open-ended questions, for being contacted for follow-up research, and for publishing the data on a depository where other researchers can request access and use the data for future research.

## Interventions

### Explanation for the choice of comparators {6b}

The intervention group will be compared to a waitlist control group. Participants in both groups will receive additional care as usual. This comparator is chosen because it is considered to be the most ethical for this study population, as the siblings in the control condition may also want to play the game.

### Intervention description {11a}

#### Serious game “Broodles”

The intervention involves playing the serious game “Broodles” (in Dutch: “Broedels”). The game was developed by the researchers in collaboration with a game development team (e.g., producer, director, artist, writer) experiential experts, healthcare professionals, and other expert researchers in the field. In addition, a co-creation process was followed with a panel of five siblings (age 6–10 years). The game is based on the Brothers and Sisters Book (Dutch: Broers- en zussenboek) by Van Dijken (ABvD) [36] and addresses the nine domains of sibling quality of life [15], using aspects of cognitive behavior therapy. The game was developed according to the design model of Derks et al. [23], and recommendations from previous studies on serious games were taken into account [20, 24]. For example, a moderate amount and duration of the levels was chosen, because in a meta-analysis fewer and shorter sessions were associated with larger intervention effects of serious games [20].

The storyline of the game evolves around the “Broodles,” which are little fantasy creatures who live unnoticed in our own world. In the game, the “Broodles” experience things that siblings of children with ID and/or VI can also experience. The stories are based on experiences shared by the siblings of the beforementioned panel and from the Brothers and Sisters Book. A special feature of the “Broodles” is that they are capable of literally putting oneself in another’s shoes for a short period of time. In this way, they can better understand their brother or sister. In each level of the game, a different pair of sibling “Broodles” is presented. Each pair consists of a child with ID and/or VI and its sibling. Throughout the game, siblings answer questions about what happens in the game, what feelings the “Broodles” might experience, how the “Broodles” can deal with the situations and the feelings, and how the stories relate to the sibling’s own experiences.

The serious game has eight levels of around 20 minutes that all have the same structure of eight game elements. The sibling will play the game by themselves, except for one level that they play with a friend. Each level discusses one domain of sibling quality of life (excluding “acceptance,” because this is more of an overall theme or aim). Each level includes (1) two animations of the “Broodles” facing and resolving a difficult situation, (2) two sets of multiple-choice questions, (3) an emotion memory game, (4) a mini documentary of five siblings sharing their own experiences, (5) a game regarding helpful and nonhelpful thoughts, and (6) a hidden object game. In addition, at the end of each level siblings receive a printable worksheet with a small offline exercise that siblings can do with their parents or friends. This worksheet can also be used to share with their parents, teachers, friends, and others the information they received concerning the theme of the level of the game. Siblings can complete the game in 4 weeks, playing two levels per week and making the worksheets in between the levels. An overview of the levels, key aims of the levels, and offline exercises can be found in Table 1.

**Table 1:** Overview of levels, key aims of the levels and offline exercises

**Level**	**Key aims of the level**	**Offline exercise**
0. Introduction	Introducing the Broodles	None, level 1 is played right after the introduction
1. Joint activities	a. Recognizing feelings about not being able to do something with the brother or sister b. Learning what can be done together with the brother or sister	Making a list of activities the sibling likes to do with his/her brother or sister
2. Mutual understanding	a. Recognizing feelings about a lack of mutual understanding b. Learning how to deal with situations when there is no mutual understanding	Interviewing his/her parents about anything the sibling wants to know about his/her brother or sister
3. Trust in wellbeing	a. Recognizing feelings of worry b. Learning that it is important to share feelings c. Learning that he/she is just as important as his/her brother or sister	Practicing helpful and nonhelpful thoughts
4. Private time	a. Acknowledging that it is OK to find your brother or sister annoying sometimes b. Learning that it is good to claim private time	Filling out a “Me-time” form and sharing it with their parents
5. Dealing with the outside world *(level played with a friend)*	a. Recognizing feelings about getting negative reactions from the outside world b. Learning how to deal with reactions from the outside world	Explaining his/her brother’s or sister’s disability to his/her friend and telling what they experience being a sibling
6. Forbearance/dealing with “different” behavior	a. Recognizing feelings about the brother’s or sister’s “different” behavior b. Learning how to deal with the brother’s or sister’s “different” behavior	Writing down when the sibling feels scared, sad, happy or mad about his/her brother’s or sister’s behavior, and sharing that with his/her parents
7. Social support	a. Recognizing feelings about not being able to do something due to the brother’s or sister’s disability b. Learning that it is good to ask for help and support	Making an overview of his/her social support network
8. Exchanging experiences	a. Knowing he/she is not the only one experiencing and having certain feelings b. Increasing awareness of sibling support groups	Writing down his/her own qualities and competences; the parent will write a compliment card

Complementing the serious game, parents receive a brochure including a short explanation of the game and how the parent can support the sibling. This also includes references to other sources of sibling support, such as books, websites, and support groups. The parent that participates in the study will be asked to read the extra information and support the sibling with the worksheets. The other parent can be involved in supporting the sibling as usual. 

Before the start of this RCT, the serious game and complementing materials were tested by seven children and their parents, and the involved group of experts. Four of these children were also involved in the development of the game and three children had never seen the game before. Of these three children, one did not have a brother or sister with special needs and the other two were above 9 years of age (respectively 10 and 11 years). Feedback was given on the attractiveness, desirability, and applicability of the intervention. Furthermore, in three cases the children were observed playing two levels, and their non-verbal behavior confirmed their evaluations. Based on their responses, minor changes were made to make the game more understandable and appealing.

### Criteria for discontinuing or modifying allocated interventions {11b}

Both siblings and parents can choose to stop playing the serious game at any time. They will then be asked if they still want to complete the assessments. From the researcher’s point of view, there are no criteria for stopping with the game.

### Strategies to improve adherence to interventions {11c}

In order to improve adherence to the intervention, the parent and sibling will receive a short verbal and written instruction about how to play the game. The parent will also receive a copy of the information brochure, which includes information about his or her role in supporting the sibling with playing the game, and about the aim and relevance of the game. In addition, the parent will receive weekly reminders that their child has to play the game. Researchers are available for questions about playing the game during these weeks.

To assess the adherence, parents will be asked at post-test assessment how many levels the sibling has played, how many worksheets have been made, how much of the information brochure the parent has read, and in which way the parent supported the sibling in playing the game and making the worksheets. The research assistant will also ask the child to show the game certificate that children only receive when they finished the game. In addition, general compliance data of the serious game will be gathered, giving insight into how many levels of the game children played.

### Relevant concomitant care permitted or prohibited during the trial {11d}

For ethical reasons, all types of care as usual are permitted during the trial. Parents are asked at all assessment points if the siblings have received professional support or used support sources. Examples of care as usual could be no care, using support sources such as books, receiving help from a social worker, or attending a sibling activity, such as a fun sibling get-together or a group training.

### Provisions for post-trial care {30}

There will be no post-trial care. No harm of participation in the trial is expected. Siblings can play the serious game again for unlimited time after the study has finished. The information brochure that parents receive also includes tips for other sibling support sources.

### Outcomes {12}

A number of standardized measures complemented with a few self-constructed questionnaires are used. Parents are asked to complete the same questionnaires and open-ended questions at three measurement points. The questionnaire about demographic variables will only be completed at pre-test assessment. Siblings are also asked to complete the same questionnaires, a drawing assessment, and open-ended questions at three measurement points. The social validity scale will only be completed by the parents and siblings at pre-test by the total group and also at post-test by the intervention group. An overview of the assessments at each measurement point can be found in Table 2.

**Table 2:** Overview of enrolment, interventions and assessments

	**Enrolment**	**Allocation**	**Post-allocation**		**Close-out**
**Timepoint**	*t*0		*t*1	*t*2	
Enrolment					
Eligibility screen	X				
Registration	X				
Informed consent	X				
Allocation		X			
Interventions					
Intervention group		X			
Waitlist control group					X
Assessments					
Demographic variables* (parent)*	X				
Control variables *(parent)*			X	X	
Quality of life *(parent and child)*	X		X	X	
Sibling adjustment to the disability of the brother or sister* (parent and child)*	X		X	X	
Self-esteem *(child)*	X		X	X	
Perceived social support *(child)*	X		X	X	
Sibling relationship *(parent and child)*	X		X	X	
Coping skills *(child)*	X		X	X	
Parent-child relationship *(parent)*	X		X	X	
Social validity *(parent and child)*	X		X		
Subjective evaluation *(parent and child)*			X		
Parenting self-efficacy *(parent)*	X		X	X	
Child behavior *(parent)*	X		X	X	

### Demographic and control variables

#### Demographic variables

The parent is asked at pre-test assessment to fill out a questionnaire about demographic variables, which is based on standard demographics and variables that were found to be important in sibling studies. The variables are (1) country (the Netherlands or Belgium), (2) gender parent that participates in the study (hereafter: parent 1), (3) relationship to child parent 1, (4) age of the parent, (5) gender other parent (hereafter: parent 2), (6) relationship to child parent 2, (7) relationship to other parent, (8) family composition, (9) work status parent 1, (10) work status parent 2, (11) education level parent 1, (12) education level parent 2, (13) household income, (14) ability to make ends meet with their household income, (15) languages spoken in the household, (16) gender child with disability, (17) age child with disability, (18) type of disability of the child, (19) comorbid disabilities of the child, (20) level of ID of the child with a disability, (21) level of VI of the child with a disability, (22) daytime activities of the child with a disability, (23) type of external care for the child with a disability, (24) nights per month that the child with a disability stays somewhere else, (25) gender sibling, (26) age sibling, (27) older or younger sibling, (28) days sibling is cared for by someone else, (29) used sibling support, and (30) used other support sibling.

#### Control variables

In addition to the demographic variables, several control variables will be included in the parent-report questionnaire at post-test and follow-up assessments in order to monitor stressful events and the utilized care as usual. Four questions can be answered: (1) changes in care or stressful events experienced by the child with a disability, (2) used sibling support, (3) used other support by the sibling, and (4) stressful events experienced by the sibling. Parents can choose between listed support sources, or name something else, and are asked to describe the experienced stressful events.

### Primary outcomes

#### Quality of life

The Pediatric Quality of Life Inventory (PedsQL 4.0) [37] is a measure of four aspects of health-related quality of life included in the subscales: Physical Functioning, Emotional Functioning, Social Functioning and School Functioning. The questionnaire will be filled out by both the parent and the sibling. Different versions are used for children aged 5–7 years and children aged 8–12 years. In this study, the Dutch version [38] of the Psychosocial Health Summary Score will be used. Specifically, the Acute version, which is filled out over the past week, will be used. The used scale has three 5-item subscales: Emotional Functioning, Social Functioning, and School Functioning. The subscale Physical Functioning is excluded, because this subscale is irrelevant for the current study. On the parent-report version and the child-report version for children aged 8–12 years, the items can be answered on a 5-point Likert scale (0 = never to 4 = almost always). On the child-report version for children aged 5–7 years, the items can be answered on a 3-point Likert scale (0 = not at all, 2 = sometimes, and 4 = a lot). An example of an item of the child-report version is: “Do you feel sad.” The internal consistency of the Psychosocial Health Summary Score was good with Cronbach’s alphas of 0.83 (child-report) and 0.86 (parent-report). Construct validity has also been demonstrated [39].

#### Sibling adjustment to the disability of the brother or sister

The Sibling Perception Questionnaire (SPQ; originally by Carpenter & Sahler [40] and adapted by Lobato & Kao [41]) measures the impact of the disability on the sibling and the sibling’s attitude towards the disability. The questionnaire has a total of 22 items on four subscales: Interpersonal, Intrapersonal, Fear, and Communication. The Dutch version [42] of both the parent-report version and the child-report version will be used in the current study. In this study, the word “illness” in the items will be replaced by “disability.” The items can be answered on a 4-point Likert scale (1 = never to 4 = often). An example of an item is: “I feel sad about the disability of my brother/sister.” In the main data analysis, the 18-item composite Negative Adjustment Scale, which is composed of the sum of the Interpersonal, Intrapersonal and Fear subscales, will be used, because a previous study [41] found that the separate subscales have low internal consistency. The internal consistency of the Negative Adjustment Scale was found to be acceptable to good with Cronbach’s alphas of 0.79 (child-report) and 0.74 (parent-report).

In addition, parents will be asked to provide a written answer to three open-ended questions about the adjustment of the sibling to the disability of the brother or sister. The siblings will be asked to verbally answer these three open-ended questions. Their answers will be audiotaped. When there is no consent for audio recording, the researcher will type down the answers of the sibling.

The questions for the siblings and parents are: (1) “Can you name up to five examples of what you/your child like(s) about your/their brother or sister?” (2) “Can you name up to five examples of what you/your child do(es) not like or find(s) difficult or unpleasant about having a brother or sister with a disability?,” and (3) “Can you explain what you/your child do(es) to deal with these examples?.” The children will also be asked to rate the examples named at question 1 and question 2 with smileys, indicating how much they like it or how hard or unpleasant they think it is. At post-test and follow-up assessments, the siblings will be asked to rate the examples named at pre-test assessment again and tell if they have changed how they deal with these kind of situations. They will also be allowed to name new examples. Parents will be asked to name what has changed in what their child likes or finds hard about having a brother or sister with a disability and how he/she deals with that. They can also name new examples.

### Secondary outcomes

#### Self-esteem

The Self-Perception Profile for Children (SPPC) [43] is a child-report scale that consists of six subscales. In order to limit the burden put on the siblings, only the Global Self-Worth subscale will be used in the current study. Specifically, the Dutch simplified 3-item version (SPPC-s) [44] of the subscale will be used, because this version is more appropriate for children aged 6–8 years. In this version, the items can be answered on a 4-point Likert scale, which is visualized with rectangles that increase in size when the answer category represents higher self-worth. An example of an item is: “Are you happy with whom you are?,” which can be answered with not happy, a bit happy, happy, or very happy. The internal consistency of the simplified 3-item Global Self-Worth subscale was acceptable with Cronbach’s alphas between 0.65 and 0.69 [44].

#### Perceived social support

The Social Support Scale for Children (SSSC) [45] is a child-report scale that consists of four 6-item subscales: Parent Support, Classmate Support, Teacher Support, and Close Friend Support. In the current study, the Dutch version [46] of the scale will be used. The items have a “structured alternative format.” The child first has to choose which of two “types” of kids they are alike, for example “Some kids have parents who don’t really understand them” or “Other kids have parents who really do understand them.” Then the child has to choose if that is "sort of true for me" or "really true for me". The items are scored from 1 to 4, with higher scores representing higher levels of perceived support. The internal consistency was good, with Cronbach’s alphas between 0.72 (Classmate Support Scale) and 0.81 (Close Friend Support Scale), and support for the validity of the scale was found [47].

#### Sibling relationship

Sibling relationship with the brother or sister with a disability is measured using two different measures. One that is completed by the sibling and one that is completed by the parent.

The Pictorial Assessment of Interpersonal Relationships (PAIR) [48] is an instrument that assesses the characteristics of a close relationship through drawings. In the current study, we use part of the instruction as described by Guidotti et al. [49]. Siblings will be asked to draw themselves with their brother or sister while doing something. The siblings receive an A4 paper, a pencil, and a set of crayons. They can take as much time to complete the drawing as they want. After they made the drawing they will be asked which figure represents whom and what they are doing. The drawings will be assessed by two independent investigators on six different scales: Cohesion, Distancing, Similarity, Value, Emotions, and Conflict. For each subscale, scores can be administered following a standard assessment procedure, which is elaborately described in the manual. The way of scoring differs per scale. In a study with siblings of children with autism spectrum disorder, the inter-rater reliability was good, with Cohen’s kappa larger than 0.90 [49]. Significant correlations between the subscales and total score, and support for discriminant validity were found [48].

The Parental Expectations and Perceptions of Children’s Sibling Relationships Questionnaire (PEPC-SRQ) [50] is a 27-item parent-report questionnaire that assesses the expectations and perceptions that the parent has of the sibling relationship of their children. On the original Perceptions scale, the parents rate the frequency, how problematic it is, how easy it is to improve, and how much they would like help with that, for each behavior. Because of the relevance to the research question, in the current study only the Perception scale will be used, and only the frequency of the behaviors will be rated by the parents. The questionnaire consists of three subscales: Warmth, Agonism, and Rivalry/Competition. At the end of the questionnaire, the parent is also asked to rate the overall quality of the sibling relationship of their children. The frequency of the behaviors is rated on a 5-point Likert scale (1 = never to 5 = always). The overall quality of the sibling relationship is rated on a 7-point Likert scale (1 = very poor, to 7 = extremely good). An example of an item is: “Loyalty or sticking up for one another.” The questionnaire was translated to Dutch by a team of researchers experienced with the population (e.g., LV) using the WHODAS 2.0 Translation package (version 1.0) [51]. The internal consistency of the original subscales is acceptable to good, with Cronbach’s alphas of 0.86 on Warmth, 0.73 on Agonism, and 0.76 on Rivalry/Competition. Support for construct validity was found [50].

#### Coping skills

The Coping Strategies Inventory (CSI) [52] originally is a 72-item questionnaire with eight primary scales, four secondary scales, and two tertiary scales measuring the types of coping strategies that children use. In order to lower the burden for the participating children, the 12-item adapted Dutch version [53] of the Engagement and Disengagement subscales of the questionnaire will be used in the current study. When completing the CSI, children are first asked to think of a difficult situation that happened in the past month. Four open-ended questions are asked to help the sibling to describe the situation. Then the child is asked to rate on a 4-point Linkert scale (1 = not at all to 4 = very much) how much the situation bothered him/her. Finally, the twelve main items about how the child reacted to this difficult situation can be answered on a 5-point Likert scale (1 = I never do this to 5 = I always do this). An example of an item is: “I blamed myself.” The validity and internal consistency of the original 72-item scale was good, with Cronbach’s alpha of. 90 for the Engagement scale, and of 0.89 for the Disengagement scale [52]. The 10-item version of Scholten et al. [53] had acceptable internal consistency for the Disengagement scale (*α* = 0.62), but insufficient internal consistency for the Engagement scale (Cronbach’s alpha is not reported). Therefore, we adapted it to a 12-item version in collaboration with one of the authors (AW).

#### Parent-child relationship

The Child-Parent Relationship Scale (CPRS) [54] is a 30-item (full version) or 15-item (short form: CPRS-SF) parent-report scale that measures positive and negative aspects of the parent-child relationship as experienced by the parent. The short form consists of two subscales: Conflicts and Closeness. In the current study, the positive aspects of the parent-child relationship are most relevant in answering the research question. Therefore, only the 7-item Closeness subscale of the short form will be used. This subscale was translated to Dutch by a team of researchers experienced with the population (e.g., LV) using the WHODAS 2.0 Translation package [51]. The items on the CPRS can be answered on a 5-point Likert scale (1 = definitely does not apply, 2 = not really, 3 = neutral, not sure, 4 = applies somewhat, and 5 = definitely applies). An example of an item is: “If upset, my child will seek comfort from me.” The internal consistency of the short-form Closeness subscale was acceptable [55], with Cronbach’s alphas between 0.64 (mothers) and 0.74 (fathers). The convergent and predictive validity of the short-form Closeness subscale was reported to be good [56].

#### Social validity and subjective evaluation

The Social Validity Scale (SVS) [57] originally is a 16-item scale to measure the desirability, feasibility, and perceived effectiveness of an intervention. The versions of the scale used in Damen et al. [58] and in Derks et al. [59] were adapted to fit the current intervention. Different versions are used for parents and siblings. At pre-test assessment, the questions are focused on the expectations about the game, at post-test assessment the questions are focused on the evaluation of the game. All parents and siblings will fill out the pre-test scale. Only parents and siblings in the experimental group will fill out the post-test scale. At the post-test assessment, additional open-ended evaluation questions are included. Items can be answered on a 5-point Likert scale, with answer categories adapted to the question (e.g., 1 = very bad to 5 = very good). The child-report version also shows smileys. Higher scores indicate more positive expectations or evaluations, with 3 being the neutral score.

At pre-test assessment, the parent and sibling will answer eight questions about the expected pleasantness, feasibility, and effectiveness. For example, one of the parent-report questions is: “I think my child can learn […] from playing the serious game ‘Broodles’.” At post-test assessment, the parent will be asked 17 and the child will be asked 23 open and closed questions about the pleasantness, perceived effectiveness, and feasibility of the serious game elements and worksheets. The open-ended follow-up questions give the parents and siblings the ability to explain their answers to the closed questions. The parent can type their answers in the provided open answer space. The answers of the sibling will be audiotaped. When there is no consent for audio recording, the researcher will type down the answers of the sibling. For example, a closed question for the child is: “How much have you learned from playing the game ‘Broodles’?” The follow-up open-ended questions are: “What is it that you have learned?,” “Which parts of the game or worksheets helped you with this?,” and “What did you miss?” In addition, the parent will also be asked five questions about the brochure with information and tips for parents.

A version of the SVS that was used in Damen et al. [60] showed good internal consistency for the subscales Feasibility (Cronbach’s *α* = 0.84) and Subjective effectiveness (Cronbach’s *α* = 0.86).

### Exploratory variables

#### Parenting self-efficacy

The Parenting Sense of Competence Scale (PSOC) [61] measures parenting self-esteem. The original scale consists of a total of 17 items on two subscales. In order to limit the burden of the parent, in the current study a 3-item adapted version of the PSOC will be used [62]. The items can be answered on a 5-point Likert scale (1 = not at all like me to 5 = completely like me). The included items are: “I feel confident in my role as a parent,” “Being a parent is manageable and any problems are easily solved,” and “I honestly believe I have all the skills necessary to be a good mother to my child.” In the current study, parents will be specifically asked to answer the question about their parenting concerning the sibling. The internal consistency of the 3-item scale was found to be good, with Cronbach’s alpha of 0.81 [62].

#### Child behavior

Six questions about the behavior of the child with a disability are answered by the parent. These questions include: (1) “My child that has a disability shows behavior that scares my child that participates in this research,” (2) “My child that has a disability shows behavior that makes my child that participates in this research happy,” (3) “My child that has a disability shows behavior that hurts my child that participates in this research,” (4) “My child that has a disability shows behavior that makes my child that participates in this research proud,” (5) “My child that has a disability shows behavior that bothers my child that participates in this research,” and (6) “My child that has a disability shows behavior that makes my child that participates in this research feel appreciated.” The parents can answer the questions on a 5-point Likert scale (0 = never, 1 = rarely, 2 = sometimes, 3 = usually, and 4 = always). The scores on the 3 items presenting negative behavior are summed, and the scores on the 3 items presenting positive behavior are summed as well.

### Participant timeline {13}

**Fig. 1:**
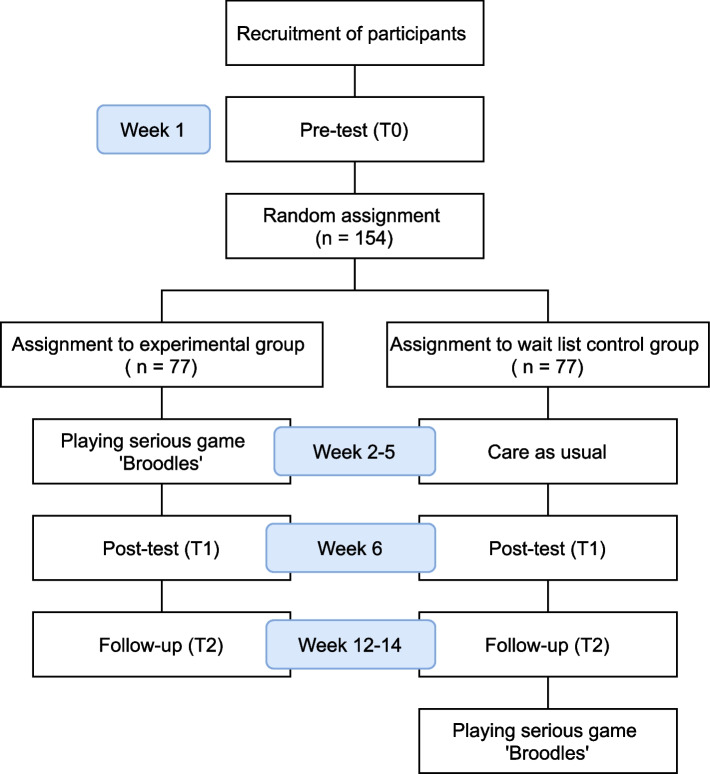
Flowchart of the study timeline

Families that show interest in participating in the study by contacting the researcher will receive the subject information letter through email. This consists of both an extensive information letter for the parent and a simplified summary for the sibling. The parent and sibling can then decide whether they want to participate in the study. When they indicate that they want to participate, the researcher will call the parent for a brief eligibility screening, to provide more information about the study procedure and to answer questions. After that the research assistant will contact the parent to plan the three home visits in which the assessments are completed. Parents are given the opportunity to choose if they want to fill out the questionnaire during the home visits or at another moment in the week prior to the visit. At the pre-test assessments, parents will hand over the informed consent forms to the research assistant prior to starting the assessment. After completing the pre-test assessments, the parent and sibling will be informed about the group they have been allocated to. The siblings in the experimental group will then play the serious game over a period of 4 weeks. The siblings in the waitlist control group can play the game after completing the follow-up assessment. A visualization of the study timeline can be found in Fig. 1.

### Sample size {14}

Using GLIMMPSE 3.0 [63], the required sample size to conduct multivariate linear mixed models for the primary outcomes quality of life and sibling adjustment to the disability of the brother or sister is calculated. The sample size is estimated with an alpha of 0.05 and a target power of 0.80 and is based on an interaction effect hypothesis, including between (intervention) and within (measurement point) variance. Correlations between measurement points are estimated to be 0.60 (T0–T1; T1–T2) and 0.40 (T0–T2), based on previously found test-retest correlations on the PedsQL [64]. The correlation between the outcomes is estimated to be 0.30. This estimate is based on a previously found correlation between the SPQ and a measure of wellbeing and adjustment (Strengths and Difficulties Questionnaire; SDQ), as no information about the correlation between de SPQ and a measure of quality of life was available [65]. A scale factor of 1.2 for variability is inserted in the sample size calculation, in order to be more conservative.

For both quality of life and sibling adjustment to the disability of the brother or sister the child-report means and standard deviations from previous sibling studies are used [41, 66]. For the control group, no change is expected on the outcome measures. For the intervention group, change is expected gradually. The expected total change (at T2) on quality of life is based on the Minimal Clinically Important Difference [67]. The expected total change on sibling adjustment to the disability of the brother or sister is based on the measured change in a previous sibling intervention study [41]. No change in standard deviations is expected on all measurement points.

The computed sample size calculation based on the beforementioned parameters results in a required sample size of 134, with 67 participants in the intervention group and 67 participants in the waitlist control group. Based on two previous intervention studies [22, 29], a dropout rate of 15% is accounted for. The total required sample size is therefore 154. This includes 154 siblings and their 154 parents.

### Recruitment {15}

Participants will be recruited from the general population. Information leaflets will be distributed among families by health care organizations and schools that provide care to children with ID and/or VI. The leaflet will contain contact addresses of the researchers. Social media and websites of care organizations, support groups, and parent networks will also be used to recruit participants. This includes information videos, pictures, and digital leaflets. Parents can also find more information on the Dutch webpage about the study.

## Assignment of interventions: allocation

### Sequence generation {16a}

The participants will be randomly allocated (1:1) individually to the experimental and control group using an allocation schedule. The randomization will be stratified by sibling age, including two stratification groups: siblings aged 6 and 7, and siblings aged 8 and 9. Stratification by sibling age is executed because we expect that the age of the sibling can influence the intervention effect. Next to stratification, block randomization will be used in order to ensure equal group sizes. The block sizes will be randomized as well, varying between 4, 6, and 8.

### Concealment mechanism {16b}

The allocation schedule will be produced and kept by an independent researcher. When a new participant is included, the researcher will ask the independent researcher what condition is allocated to that participant’s number. The researcher will then send an email with this information to the research assistant that will visit the participant for the assessment. The research assistant will be requested to open this email after the participant has completed the pre-test assessment.

### Implementation {16c}

The researcher who enrols the participants will chronologically assign a number to each participant based on the moment of entry. The allocation schedule will be produced with a computerized random number generator by an independent researcher, and not be visible for the researchers.

## Assignment of interventions: blinding

### Who will be blinded {17a}

Participants and research assistants will only be blinded for intervention at the pre-test assessment. The researchers that execute the data analysis will be blinded for intervention. The assigned intervention will not be visible in the dataset.

### Procedure for unblinding if needed {17b}

This is not applicable, as participants and investigators are no longer blinded after pre-test assessment.

## Data collection and management

### Plans for assessment and collection of outcomes {18a}

Siblings and parents will complete 60- to 90-min assessments at pre-test (T0), post-test after 5 weeks (T1), and follow-up, 6 to 8 weeks after the post-test (T2). In total, the data collection takes 12 to 14 weeks per participant. Siblings and parents will separately fill out questionnaires on a computer or tablet using Qualtrics [68]. Some of the outcomes are measured with both parent- and child-report questionnaires, whereas other outcomes are measured with only one informant.

Research assistants will visit the participants at their homes to take all assessments following a standard protocol. These assistants are Bachelor or Master students in educational sciences at the Vrije Universiteit Amsterdam. The students will receive a 2-hour training on taking the assessments by the researcher. During the home visits, the research assistant will give instructions to the siblings and read out the questions. Parents can fill out the questionnaire without assistance during the home visit, or at another moment in the week prior to the visit. However, the research assistant can provide the parent, when needed, with an extra standard explanation about the questions.

### Plans to promote participant retention and complete follow-up {18b}

In order to promote participant retention, the research assistant will remain contact with the parent during the study period. The home visit for the follow-up assessment will already be planned at the end of the post-test assessment. This home visit can be planned within a timeframe of 3 weeks, to make it more convenient for the family to choose an appropriate moment. The research assistant will remind the parent of this home visit 1 week before it takes place. No additional outcome data will be collected from participants that no longer want to complete the assessments.

### Data management {19}

Based upon entry, a participant number will be assigned to all participants (consisting of pairs of a parent and a child) by the researcher. The key-file will contain the code, name of the parent, name of the child, email address of the parent, phone number of the parent, and address of the family. The key-file will be stored separately from the research data on a secured cloud-based shared-storage environment on the server of the Vrije Universiteit Amsterdam, and will only be accessible to the researcher, principal investigator, and monitors. The research assistants that are involved in data collection will get access for a limited time to only a personal folder on the secured storage environment where they can find the contact information and participant number of the participants they visit.

Most of the research data will be collected using the safe online survey software Qualtrics. In Qualtrics, only the participant number will be entered, there will be no data that makes direct identification of the participant possible. In addition, audio recordings and drawings are obtained. Only the participant number will be written on the drawings that the siblings will make. Digital game statistics, including the user name of the participant but no other identifying information, are obtained as well as the children play the game on a secured website on the server of the Vrije Universiteit Amsterdam. Research data will be saved encrypted and processed using an identification number on a secured cloud-based shared-storage environment on the server of the Vrije Universiteit Amsterdam with automatic back-up. Paper files will be stored in locked cabinets.

After publication, the anonymized dataset of the quantitative data will be made available upon request for future research for 15 years on an appropriate data archive. Personal data and informed consent forms will be archived in a safe depository for 15 years as well. A data management plan has been composed with assistance of a data management expert.

### Confidentiality {27}

Researchers and student who are involved in collecting and processing data will sign a nondisclosure agreement. Other persons that have access to the research data include the review committee; the security committee who monitor the data for research purposes; and the persons who check the quality of the study. These persons will also sign a nondisclosure agreement. Data will be handled confidentially and in accordance with the General Data Protection Regulation (GDPR), in order to ensure the privacy of the participants. All research data is pseudonymized.

### Plans for collection, laboratory evaluation, and storage of biological specimens for genetic or molecular analysis in this trial/future use {33}

No biological specimens are collected in this study.

## Statistical methods

### Statistical methods for primary and secondary outcomes {20a}

In the current study, quantitative data will be analyzed using IBM SPSS Statistics 27 [69], and qualitative data will be analyzed using ATLAS.ti. 22 [70].

The primary and secondary objectives, as well as the exploratory analysis on parenting self-efficacy, will be answered by computing the linear mixed-effects model procedure (MIXED). To test the effect of the intervention, the main effects of intervention and time, together with the interaction effect between intervention and time, will be included in the model. The primary objectives will be analyzed multivariate, whereas the secondary objectives are analyzed univariate. For the latter, the Bonferroni correction will be used to determine the alpha level. Child-report and parent-report outcomes will be analyzed separately. Cohen’s Ds will be calculated to determine the effect size. The answers to the open-ended questions will be analyzed using thematic coding with conventional qualitative content analysis [71]. The outcomes of the PAIR will be analyzed in both a quantitative way using the manual [48]. Thematic analysis of the drawings will also be executed according to the procedure described in Guidotti et al. [49]. The drawings of the PAIR are scored by two independent researchers. Intra-class correlations will be calculated to assess the inter-rater reliability.

For social validity, the mean scores will be reported. Additionally, one sample *t*-tests will be executed in order to test if the mean score significantly differs from the neutral score (3). Conventional qualitative content analysis will be used to analyze the answers to the open-ended evaluation questions [71].

### Interim analyses {21b}

Interim analyses are not planned beforehand. However, when after 1 year not more than half of the required sample size is met, interim analyses will be considered and executed by independent investigators. The power calculation will be executed again in order to determine if the required sample size will be doable and the study should or should not be continued.

### Methods for additional analyses (e.g., subgroup analyses) {20b}

Descriptive statistics and Pearson correlations between outcome measures are calculated. The data will be checked for outliers, which will be winsorized when found. Intervention uptake will be reported, using the percentage of the levels and worksheets that have been completed, and the part of the parent brochure that has been read. The two conditions (intervention and control group) will be compared based on baseline scores. When significant baseline differences between the conditions occur on demographics or outcome measures, and these variables are associated with the outcome measure, this will be controlled for in further analyses. Sensitivity analysis will then be executed.

When the data regarding the type of disability allows it (i.e., the group sizes are large enough), the differences in outcome measures at T0 between siblings with a brother or sister with different types of disabilities will be analyzed as well using ANOVA.

Moderation effects will be calculated using linear mixed-effect models as well, by including the moderator, and the two- and three way interaction terms with condition and time.

### Methods in analysis to handle protocol non-adherence and any statistical methods to handle missing data {20c}

Intention-to-treat analysis will be executed. All participants that are randomized, including treatment dropouts and study dropouts will be included in the main analysis. Maximum likelihood estimation will be used for missing data. In order to determine the robustness of the results, two sensitivity analyses will be conducted: one with the data of all participants that completed all assessments and the intervention, and one with the data of all participants that completed all the assessments but not the intervention (treatment dropouts). Participants will be considered a treatment dropout when they did not complete the game. When a parent has not (fully) read the complementary information brochure, this is not considered as a treatment dropout. The latter also accounts for not making all the complementary worksheets. In addition, the number of played levels will be included as a moderator in the analyses.

### Plans to give access to the full protocol, participant-level data, and statistical code {31c}

The full protocol is available upon reasonable request. The anonymized dataset of only the quantitative data and the statistical code will be accessible for other researchers upon request. Data requests will be reviewed by the researchers and data sharing agreements will need to be signed.

## Oversight and monitoring

### Composition of the coordinating center and trial steering committee {5d}

This trial is executed in a single research centre, namely the Vrije Universiteit Amsterdam. The research team in this trial consists out of prof. dr. P.S. Sterkenburg, dr. A.M. Willemen, S.D.M. Derks MSc, and L.K.M. Veerman MSc. The research team meets every other week. The coordination of the trial is led by L.K.M. Veerman, who is a PhD student on this project. This includes coordinating the recruitment and data collection, training research assistants, data management, and data analysis. Prof. dr. P.S. Sterkenburg has the final responsibility, as she is the principal investigator.

### Composition of the data monitoring committee, its role and reporting structure {21a}

A data monitoring committee (DMC) is not needed, as participation in the study does not have risks for the participants.

### Adverse event reporting and harms {22}

It is not expected that any adverse events will take place during this study. There are no risks. However, when unexpected adverse events take place, these will be reported to the medical-ethical committee (METc VUmcC).

### Frequency and plans for auditing trial conduct {23}

This study is embedded in the research institute Amsterdam Public Health (APH; reference: SQC2022-023). Part of the APH quality control of research projects is to randomly select a study for an audit. The study will be conducted according to APH standards.

### Plans for communicating important protocol amendments to relevant parties (e.g., trial participants, ethical committees) {25}

Important protocol modifications will be reported to and need to be approved by the medical-ethical committee (METc VUmc). When modifications are approved, these will also be reported to the participants, and be added to the Trials paper and the study registration on ClinicalTrials.gov.

### Dissemination plans {31a}

The outcomes of this study about the effectiveness of the intervention will be reported in article(s) in international peer-reviewed journals. Both positive and negative outcomes will be reported. The outcomes will also be reported in professional magazines in the field and in magazines for parents. The results will be presented in participating care organizations and at scientific conferences.

## Discussion

The support for siblings of children with disabilities is scarce and fragmented, even though studies have shown that these siblings can benefit from support [6]. Although several interventions for siblings have been developed, these are costly and time-consuming and the effects have not been studied with randomized controlled trials. This study will investigate the effectiveness of the first serious game for siblings (aged 6–9 years) of children with ID and/or VI, using an RCT design.

The serious game, named “Broodles,” is an educational game that siblings can play without assistance of an adult. The game consists of eight 20-minute levels that all have the same structure of eight game elements. The serious game aims to improve sibling’s quality of life, adjustment to their bother’s or sister’s disability, and multiple aspects of psychosocial wellbeing, through helping them to better understand and deal with thoughts, feelings, and difficult situations.

The expectation is that the current study will contribute to the development of support sources for siblings of children with disabilities. If the outcomes of this study show that the serious game is effective, the game will be readily available to all siblings through an open webpage. This gives parents, teachers, and caregivers tools to support siblings. Because one can never know the impact of the care burden on the family, due to different social and additional circumstances, and a care system that has only been focused on the child with a disability, it is first of all important for the game to be free of charge and that siblings are able to play it without assistance. And yet, as siblings belong to the family context and all members have an effect on each other’s wellbeing, the serious game could, when proven to be effective, preferably be offered by care organizations as part of a family intervention. This can be supportive because, based on clinical practice (in the Netherlands and Belgium), it has become clear that there is a lack of structural acknowledgment of siblings’ needs and that siblings are often forgotten when offering family interventions. The serious game may raise more awareness of the role, and thoughts and feelings of siblings. It can also be a referral to other and more intensive support sources for siblings who need extra help. Also, the close collaboration with care organizations can contribute to the implementation of the serious game in practice.

Additionally, this study will contribute to the knowledge about siblings’ support needs and sibling interventions. Few randomized controlled trials have been executed in the field of serious games for children and the field of sibling interventions [16–18, 20]. Besides, the qualitative measures may provide more insight into which aspects of the intervention siblings and parents experience as helpful. This could give direction to future research about this or other interventions for siblings. Finally, this study will be the first to specifically examine an intervention focusing on contributing to the well-being of siblings of children with VI.

Previous research shows that it remains unclear whether a sibling intervention should be offered to siblings of children with a specific diagnose type or not [16]. In order to develop a game that addresses situations that siblings can relate to the most, we did select a specific group for this study. However, when the game is found to be effective for this population, it can be investigated if the game is also effective for a broader group of siblings.

In conclusion, siblings of children with disabilities should also be supported, although there are only a few evidence-based interventions. The present study will provide insight into the effectiveness of the newly developed serious game “Broodles” in improving quality of life, adjustment to the disability of the brother or sister, and multiple aspects of psychosocial well-being of siblings of children with ID and/or VI. If this first serious game is proven to be effective, it can be used as a free of charge, easily accessible, preventive intervention for siblings.

## Trial status

Recruitment started on March 22, 2022, in the Netherlands and Belgium, and the first participant was enrolled on April 22, 2022. The first assessment started on April 22, 2022. Recruitment will continue until the required sample size is met, or at a maximum up until December 2023.

Protocol version 1, May 2022

## Data Availability

The pseudonymized dataset of the quantitative data will be made available upon reasonable request at DataverseNL.

## Abbreviations

APH: Amsterdam Public Health
CPRS: Child-Parent Relationship Scale
CSI: Coping Strategies Inventory
GLIMMPSE: General Linear Multivariate Model Power and Sample Size
ID: Intellectual disability
METc: Medical Ethics Committee
PAIR: Pictorial Assessment of Interpersonal Relationships
PedsQL: Pediatric Quality of Life Inventory
PEPC-SRQ: Parental Expectations and Perceptions of Children’s Sibling Relationships Questionnaire
PSOC: Parenting Sense of Competence Scale
RCT: Randomized controlled trial
SPPC: Self-Perception Profile for Children
SPSS: Statistical Package for the Social Sciences
SPQ: Sibling Perception Questionnaire
SSSC: Social Support Scale for Children
SVS: Social Validity Scale
VI: Visual impairment
VUmc: Vrije Universiteit Medical Center
WHO: World Health Organization
